# Keep calm and don’t stop growing: Non-consumptive effects of a sympatric predator on two invasive Ponto-Caspian gammarids *Dikerogammarus villosus* and *Pontogammarus robustoides*

**DOI:** 10.1371/journal.pone.0182481

**Published:** 2017-08-03

**Authors:** Łukasz Jermacz, Jarosław Kobak

**Affiliations:** Department of Invertebrate Zoology, Nicolaus Copernicus University, Toruń, Poland; Consejo Superior de Investigaciones Cientificas, SPAIN

## Abstract

Predators shape prey populations by elimination of individuals (consumptive effects) and by inducing modifications in prey behaviour, physiology or morphology (NCE—non-consumptive effects). Due to the resource allocation to defence, decreased feeding and higher stress, the costs of predator NCEs can be considerable. Therefore, the resistance to NCEs may be crucial for population growth and interspecific competition. We tested the resistance of Ponto-Caspian gammarids *Dikerogammarus villosus* and *Pontogammarus robustoides* to NCEs imposed by their predator, the racer goby *Babka gymnotrachelus*. As *D*. *villosus* is often avoided by predators in the presence of alternative food, we hypothesised that it would bear lower behavioural and physiological costs of anti-predator responses. We tested gammarid feeding in short-time experiments (2–4 h) with food (chironomid larvae) located at various distances from the stony shelter (to enforce food searching, Experiment I) or in the direct gammarid proximity (no searching needed, Experiment II). Moreover, we checked the predator effect on gammarid growth in a 2-week Experiment III. Both gammarids exposed to predators reduced feeding efficiency outside the shelter (Experiment I). Contrary to our expectations, the response of *D*. *villosus* was stronger. When food was provided in their direct proximity (Experiment II), the feeding of both species was unaffected by predators, indicating that a shelter supplied with food can reduce predator NCEs. The growth of *P*. *robustoides* was reduced in the presence of predators (Experiment III), whereas that of *D*. *villosus* was unaffected. Although *D*. *villosus* has a more effective defence strategy than *P*. *robustoides*, it bears similar or even higher behavioural costs of NCEs. However, it exhibits the higher resistance to the long-term predator presence, sustaining its growth rate under such conditions. This may be one of the factors contributing to the great invasion success of *D*. *villosus*, currently taking place in European fresh waters.

## Introduction

Sustained interaction between predator and prey species is a fundamental force driving changes in community structure and dynamics in every ecosystem. Predators shape prey populations doubly, by consumption of the most vulnerable individuals (consumptive effects—CEs) and indirectly by inducing costly defensive changes in development, morphology, physiology and behaviour (non-consumptive effects, also called trait-mediated effects or non-lethal effects—NCEs) [1]. So far, direct prey consumption was a subject of numerous theoretical and empirical studies helping understand the dynamics of interaction between prey and predatory species. During recent years some studies showed the costs of non-consumptive effects to be equal or even exceed those of the direct predation, significantly affecting the functioning of the community [1–4]. Predation risk alone induces physiological stress [5], increases susceptibility to pathogens [6,7] and reduces nutritional status [8], and as a consequence causes nonconsumptive mortality [9]. In contrast to direct predation, when relatively few individuals are killed by the predator, the cost of non-consumptive effects affects all members of the community [10], especially in an aquatic environment [2]. Therefore, resistance to negative aspects of non-consumptive effects may positively affect survival, population growth and the outcome of interspecific competition [11].

Studies focused on the factors determining the strength of non-consumptive effects are rare and often limited to predator traits, such as predation mode [12], predator density [13] and predator size [14]. However the strength of NCEs is also related to prey defence capabilities [15]. Generally, the magnitude of NCEs depends on CE: the stronger predation, the higher cost of anti-predator defence. Therefore it is likely that species not preferred by predators have lower defence costs than those experiencing strong predation. According to the increased competitive ability hypothesis [16], alien species which have escaped from native predator pressure and are less affected by local, naïve predators reallocate available resources from defence mechanisms into growth and development. This may give them advantage over their native competitors, susceptible to local predators.

Invasive species are major cause of the global decline in biodiversity due to competition [17], predation [18], novel pathogen transmission [19] and habitat modification [20]. However, to be successful, invasive species need features providing them with an advantage over their native counterparts, such as opportunistic feeding strategy [21,22], effective reproduction [23] or higher tolerance to abiotic factors [24]. One of the basic barriers protecting native ecosystems against invasions are native predators [25]. However, growing evidence shows that invasive species are able to recognize and effectively respond to novel predators [26,27]. Moreover, some invaders are less favoured prey for both native and invasive predators [28]. In such situations, the cost of non-consumptive predator effects should be significantly lower than that experienced by species suffering higher predation pressure.

In European waters, Ponto-Caspian invertebrates belong to the most successful invasive species. The most dangerous species among them are *Dikerogammarus villosus* [29] and *Pontogammarus robustoides* [30]. Both are relatively large and omnivorous, with highly variable trophic niches [31], but commonly exhibited preferences for food of animal origin, [18,30]. They usually displace native gammarids to suboptimum habitats or cause their local extirpation [32–34]. *Dikerogammarus villosus* is recognized as a “killer shrimp”, capable of eliminating other gammarids, both native and exotic, including *P*. *robustoides* [18,35]. Substratum preferences of the tested species are quite similar: both prefer hard and complex substrata, which leads to direct competition and displacement of the weaker species [35,36]. These species experience different predator pressure. For example, *D*. *villosus* is avoided by fish compared to other gammarids, due to its harder exoskeleton and lower nutritional quality [37]. Moreover, *D*. *villosus* was found to utilize shelters more effectively than other gammarids [38]. Therefore, *D*. *villosus* was even found to be attracted by the scent of a predator sharing its diet, despite the potential risk [26]. All these studies indicate the high resistance of this species to direct predation and suggest its resistance to non-consumptive effects.

The aim of the present study was to examine the impact of a predator cue on the feeding behaviour and growth of these two invasive gammarids under experimental conditions. The working hypothesis was that *D*. *villosus*, as a species avoided by predators, would exhibit less pronounced responses to predation cues, which would allow it to sustain higher feeding and growth rates under predation pressure, than *P*. *robustoides*.

## Material and methods

Both gammarids were captured with a hand net from the sandy or rocky bottom of the littoral zone of the Włocławek Reservoir on the lower River Vistula, Central Poland (N: 52°37’03”, E: 19°19’37”) in April 2016. After transport, the gammarids were kept in 100-L single species stock tanks with a filtration and aeration system. Tank bottoms were covered by natural substratum, such as pebbles and zebra mussels, providing optimal shelters for gammarids. Gammarids of 1–1.5 cm in length were randomly selected and attributed to the experimental treatments described below after acclimation to experimental conditions, at least 5 days but not later than 2 weeks after capture. Each individual was tested only once.

As a predator, we used the racer goby *Babka gymnotrachelus* (Kessler, 1857), also a Ponto-Caspian invasive species, for which gammarids are an important element of the diet in their native [39] and alien range [40]. The fish were collected at the same time and from the same localization as gammarids by electrofishing (type EFGI 650, BSE Bretschneider Spezialelektronik, Germany). After transport, the fish were placed in 100-L stock tanks (5 fish per tank) with aeration and filtration systems and with artificial shelters (1 per fish) to reduce competitive tension among individuals. The fish were used only once within each experiment, but they were re-used among the experiments to avoid capturing and keeping an excessive number of fish, as required under the permission from the Local Ethics Committee.

Water in all the stock tanks was regularly changed (30% of the water volume once a week). Gammarids and gobies were fed daily with frozen chironomid larvae (5 and 40 mg per individual, respectively). The physicochemical parameters (temperature 19°C, sustained by air conditioning, conductivity 480–530 μS/cm) were checked regularly using a multimeter Multi340i (WTWGmbH, Weilheim, Germany). During the acclimation time and experiments we did not observe any negative consequences of transport or stock conditions. The collection of fish and the experiment were conducted under permit of the Local Ethics Committee in Bydgoszcz, Poland, statement no 35/2013 from 12 December 2013.

### Experiment I—The impact of predator and food availability on the feeding efficiency of invasive gammarids

The experiment was conducted in 22.5-L experimental tanks. The tanks were separated into two equal parts with a mesh (1 mm) barrier. In one part, we placed a single fish (predator treatment) or left it empty (control). Before (48 h) and during the experiments the fish were fed with gammarids, which simulated the highest predation risk due to emitted predator kairomones and gammarid alarm signals [26,35]. In the test, we used live fish constantly present in the experimental tanks to guarantee the continuous presence of the predation signal during the entire experiment (4 h). A previous study demonstrated that predator kairomones were active for at least 3 h, but after 6 h did not induce the antipredator response [41].

**Fig 1:**
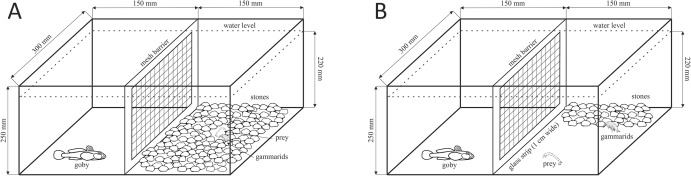
Experimental setup for Experiment I to test the effect of the predator presence and food proximity on the consumption of chironomids by gammarids.

**Fig 2:**
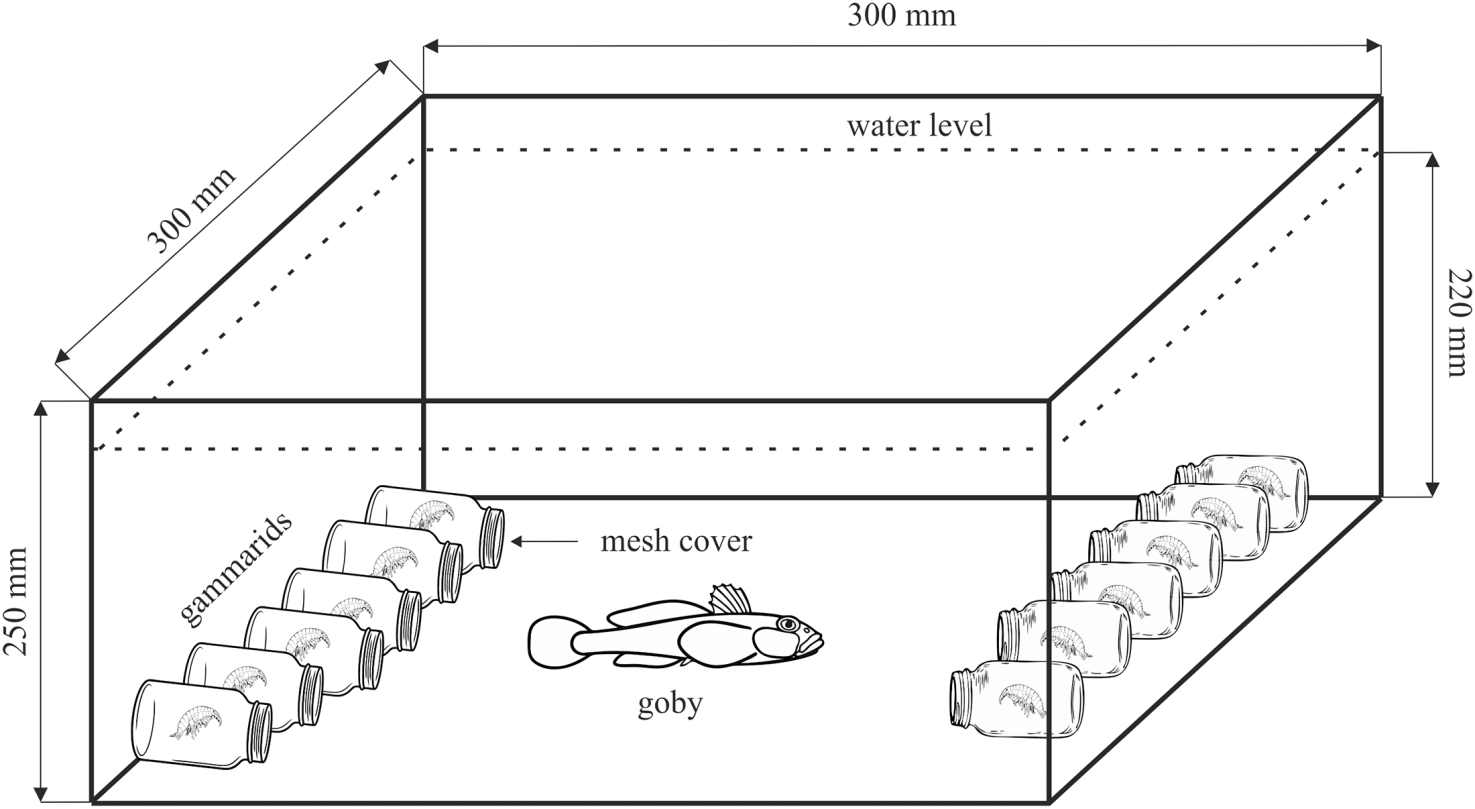
Experimental setup of Experiment III to test the effect of the predator presence on gammarid growth.

Five individuals of a single gammarid species were placed in the other part of the tank. They could perceive signals emitted by the fish consuming their conspecifics behind the mesh barrier in the predator treatment. The gammarids were tested in the presence of: 1) stones (shelters) covering 90% of the tank bottom and food located on the open bottom in the direct proximity of the shelter (2–3 cm, Fig 1A) and 2) stones covering 40% of the tank bottom and food placed on the open bottom 12–15 cm from the shelter (Fig 1B). We varied the distance to the food source to check the effect of the level of predation risk experienced by feeding gammarids. Altogether, there were four treatments: with and without predation cues and with two distances from shelters to the food source. Each treatment was replicated 12 times. We used living chironomid larvae (1 individual per gammarid) as food. During the test we did not observe the movement of chironomid larvae outside the feeding ground. Chironomids are a natural element of the diet of the tested gammarids [30]. We used stones of length 27 ± 4.8 mm, which are preferred by gammarids as shelters [36,42]. Duration of the experiment was 4 hours, starting from placing the gammarids in the tanks. The experiment was recorded using an IP video camera (SNB-6004, Samsung, South Korea) placed above the tanks. To standardize the hunger level, gammarids were not fed for 24 hours before the experiment.

Recorded material was analysed to determine (1) the number of eaten chironomids and (2) time spent in the feeding ground (outside the shelter). After catching prey gammarids often returned to the shelter where they were not visible. Therefore, the chironomids which disappeared from the feeding ground were considered as eaten. Additionally, to confirm the number of eaten chironomids, the stones were removed from the tanks after each test and searched for the presence of remaining larvae. Time spent in the feeding ground (outside the shelter) was determined with Noldus Ethovision XT 10.1 software.

We compared the counts of eaten chironomids among gammarid species (*D*. *villosus*, *P*. *robustoides*), food availability (food resources distant or close to the shelter) and predator presence (a fish present or not) using a 3-way Generalized Linear Model with Poisson distribution and log link function. Time spent outside the shelter (log-transformed) is strongly related to the surface of the open area, therefore each of the shelter treatments was analysed independently using a 2-way ANOVA (with fish presence and gammarid species as factors).

### Experiment II—The effect of predator on gammarid consumption and feeding rate

To check the impact of predation risk on the details of consumption behaviour (hidden in Experiment I) at a microscale, single gammarids were placed in 100 ml cylindrical tanks, filled to 3/4 with clean and aerated water. To standardize their hunger level, all individuals were put in the experimental tanks 24 h before the test. After this time, 30 ml of water containing predator kairomones was added to half of the tanks, while clean water was added to the remaining tanks. The previous studies demonstrated that the main source of information about predator presence for gammarids consists of chemical signals [26,41,43]. The water with predator kairomones was prepared by incubation of 5 gobies in a 100-L tank. During the incubation, gobies were fed daily with the same gammarid species as used in the test for 3 days [26]. After that, the fish were starved for 24 h (to avoid any scents of unconsumed food items in the signal water) and the water was taken for the test. The predation cue produced according to this procedure was proven to trigger the responses of the same gammarid species in an earlier study [26]. Five minutes after adding the predation cue, we placed 2 living chironomid larvae in the tank. The duration of the experiment was 2 hours. It was replicated 40 times for each treatment and species. It was demonstrated that even 3 h after receiving the predator signal, it induced a self-defence response of gammarids [41]. The experiment was recorded using an IP video camera (SNB-6004, Samsung, South Korea) placed above the tanks.

Using the recorded material, we manually determined: (1) amount of chironomids eaten (percentage of the total amount offered) and (2) feeding rate (amount of chironomids eaten per time spent eating). The amount of consumed chironomids was estimated on the basis of length difference observed in photographs taken at the beginning and at the end of the test. A gammarid was assumed to be eating when it stayed in a direct physical contact with its prey.

The percentages of chironomids eaten were not normally distributed and no transformation was able to change that. Therefore, we compared this variable between the treatments with and without predators (separately for both gammarid species) and between gammarid species (separately for each predator treatment) with a non-parametric Mann-Whitney test. The feeding rate (log-transformed) was compared among gammarid species and treatments using a two-way ANOVA.

### Experiment III—Impact of predator on gammarid growth

To test gammarid growth, we kept 12 gammarids of a single species in an aerated 22.5-L tank in the presence or absence of a single goby (Fig 2). Average initial weight of tested gammarids was 57.98 mg (*D*. *villosus*) and 37.54 mg (*P*. *robustoides*), which corresponded to natural differences in size between the species. Initial weights of the particular species did not differ significantly among experimental treatments (t-test: *D*. *villosus*: t_112_ = 1.20, p = 0.231; *P*. *robustoides*: t_86_ = 1.89, p = 0.062). Each gammarid was placed in a separate circular container (height: 7 cm, diameter: 5,5 cm) with a mesh cover to avoid cannibalism. Each container contained 2–3 pebbles as shelters [36,42]. To provide optimal water circulation, the containers were placed horizontally in the tank. During the test, gammarids were fed once a week with 20 individuals of living chironomid larvae and 5 cm^2^ of alder leaf detritus. Preliminary trials showed that this dose was sufficient to meet daily nutrition requirements of the gammarids and the presence of detritus significantly increased their survival. This corroborates the field observations, showing the consumption of plant food by gammarids [31]. The predator pressure was simulated by a single fish, freely swimming among the containers with gammarids and fed daily with living gammarids of the same species as the one being tested to increase the stress conditions. The fish were absent from the control tanks. Gammarids were weighed to the nearest 0.1 mg with a Radwag AS 110/C/2 laboratory scales (Radom, Poland) at the beginning and at the end of experiment, after two weeks of exposure. The experiment was replicated 8 times for each species and treatment.

To check for the differences in gammarid growth among the treatments, we applied a three-way nested ANOVA with Predator presence and Gammarid species as fixed factors and Tank as a random factor nested in the others. We used percentage weight changes between the initial and final measurements as a dependent variable.

### General remarks on statistical analyses

The data were checked for normality and homoscedascticity with Shapiro-Wilk and Levene test, respectively, and transformations were applied to meet these assumptions as needed. Significant effects detected in the conducted analyses were further examined with sequential Bonferroni-corrected Fisher LSD tests as a post hoc procedure.

## Results

### Experiment I

#### Feeding effectiveness

**Fig 3:**
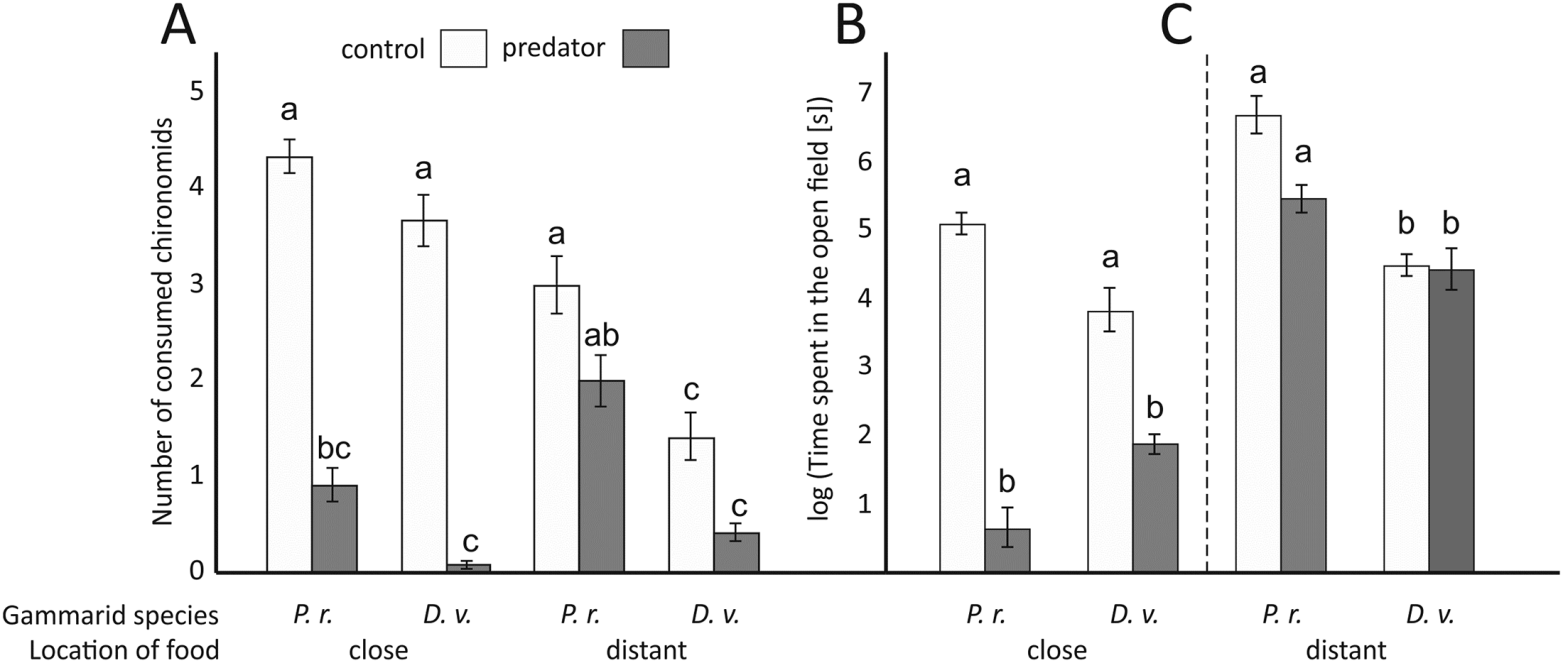
The effect of the predator presence and food proximity (Experiment I) on the mean numbers of chironomids consumed by *Dikerogammarus villosus* (D.v.) and *Pontogammarus robustoides* (P.r.) (A) and mean times (log transformed) spent by the gammarids in the open field (B, C). Treatments labelled with the same letter do not differ significantly (P > 0.05) from one another. Error bars indicate standard errors of the mean.

The number of consumed chironomids depended on interactions between distance to the food source and predator presence as well as between gammarid species and predator presence (Table 1). Gammarids significantly reduced consumption in the presence of the predator, but the reduction was significant only when the food source was located at a shorter distance from the shelter (Fig 3A). *D*. *villosus* exhibited a stronger response to the predator presence than *P*. *robustoides*. When the food source was distant from the gammarid shelters, the reduction in their feeding was much lower and non-significant. Under the control conditions, *D*. *villosus* consumed significantly less distant food compared to the close food resources. Also, when the food source was distant, *P*. *robustoides* consumed significantly more chironomids than *D*. *villosus*, independently of predator presence.

**Table 1:** The 3-way Generalized Linear Model analysis to test the effect of the gammarid species, presence of predators, and food proximity on the percentage of chironomids consumed (Experiment I).

Effect	df	Wald Chi^2^	P
Species (S)	1	15.596	<0.001*
Predator (P)	1	32.271	<0.001*
Distance (D)	1	0.670	0.413
S x P	1	5.935	0.015*
S x D	1	0.015	0.901
P x D	1	8.961	0.003*
S x P x D	1	1.180	0.277

Asterisks indicate statistically significant effects.

#### Time spent outside the shelter

Time spent by gammarids in the open field under conditions of direct food proximity depended on gammarid species and predator presence, as showed by a significant interaction in the ANOVA (Table 2A). In the presence of fish, both gammarids significantly reduced time spent in the open space, though the response of *P*. *robustoides* was clearly stronger than that of *D*. *villosus* (Fig 3B). Time spent by gammarids in the open space, when food resources were located further from their shelter only depended on gammarid species (Table 2B). Compared to *D*. *villosus*, *P*. *robustoides* spent significantly more time outside the shelter (Fig 3C).

**Table 2:** Two-way ANOVAs to test the effect of the gammarid species and presence of predators on the time spent in the open field (Experiment I) in the direct proximity of food to the shelter (A) and when food was located further away from the shelter (B) (*).

	Effect	df	MS	F	P	η^2^
A. Food in the direct proximity of the shelter	Species (S)	1	0.087	0.029	0.865	0.001
	Predator (P)	1	105.782	35.717	<0.001*	0.472
	S x P	1	14.916	5.037	0.030*	0.112
	Error	40	2.962			
B. Food distant from the shelter	Species (S)	1	31.191	11.471	0.001*	0.207
	Predator (P)	1	4.160	1.530	0.223	0.034
	S x P	1	5.044	1.855	0.180	0.040
	Error	44	2.719			

Asteriskts indicate statistically significant effects.

η^2^ values indicate the effect sizes for particular terms.

### Experiment II

**Fig 4:**
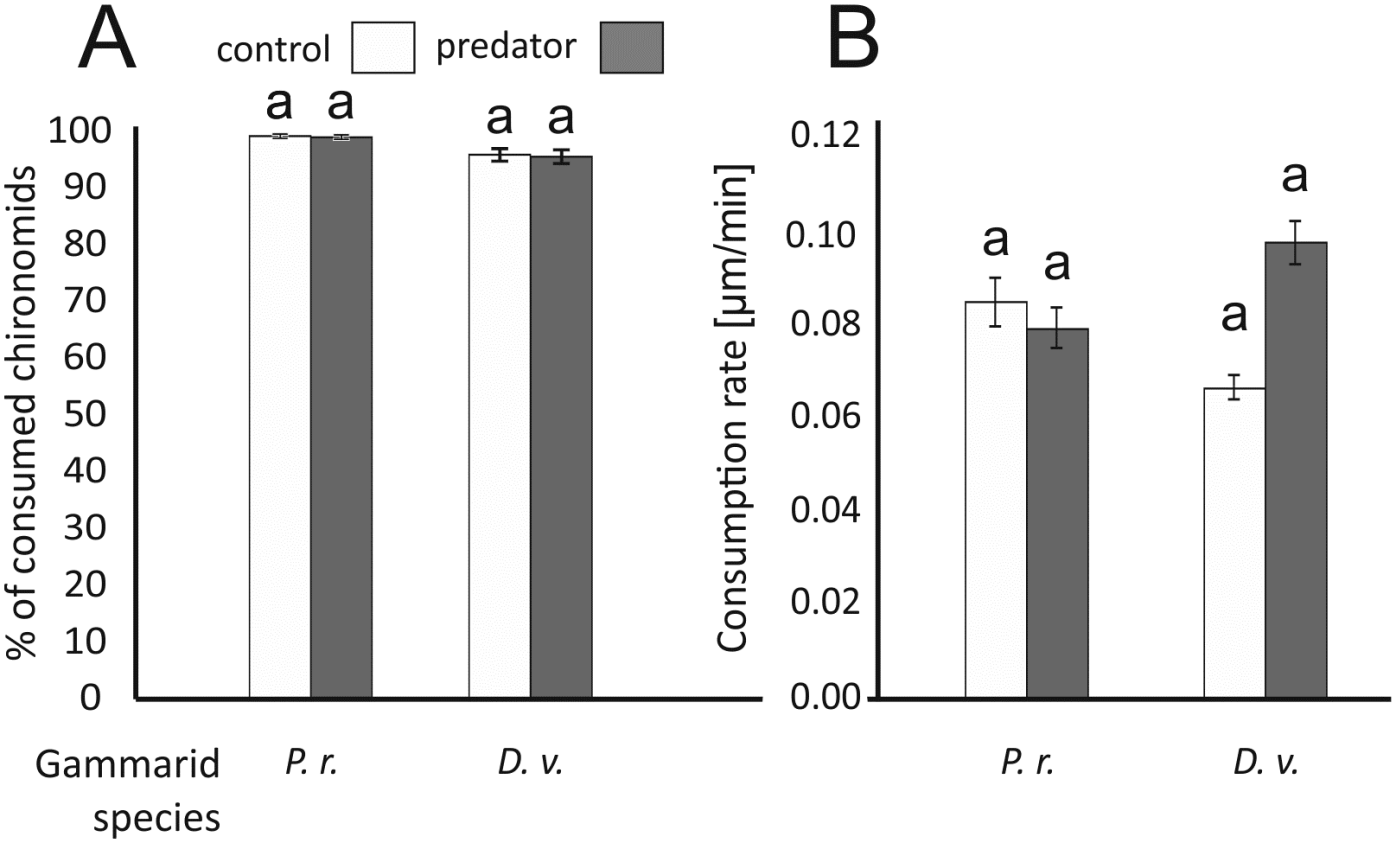
The effect of the predator presence on the mean percentages of consumed chironomids (A) and consumption rates (B) by *Dikerogammarus villosus* (D.v.) and *Pontogammarus robustoides* (P.r.) in Experiment II. Treatments labelled with the same letter do not differ significantly (P > 0.05) from one another. Error bars indicate standard errors of the mean.

The amount of consumed chironomids (Fig 4A) was not affected by predator presence (Mann–Whitney U tests: for *D*. *villosus*: Z = -0.05, P = 0.959; for *P*. *robustoides*: Z = -0.17, P = 0.867) nor by gammarid species (Mann–Whitney U tests: for control conditions: Z = -1.22, P = 0.222; in the presence of predator cues: Z = -1.05, P = 0.292). Gammarid feeding rate was also independent of the predator presence and gammarid species (Table 3; Fig 4B).

**Table 3:** Two—way ANOVA to test the effect of the gammarid species and presence of predators on the food consumption rate (Experiment II).

Effect	df	MS	F	P	η^2^
Predator (P)	1	0.522	2.038	0.157	0.023
Species (S)	1	0.052	0.205	0.652	0.002
P x S	1	0.747	2.918	0.091	0.032
Error	87	0.256			

η^2^ values indicate the effect sizes for particular terms.

### Experiment III

**Fig 5:**
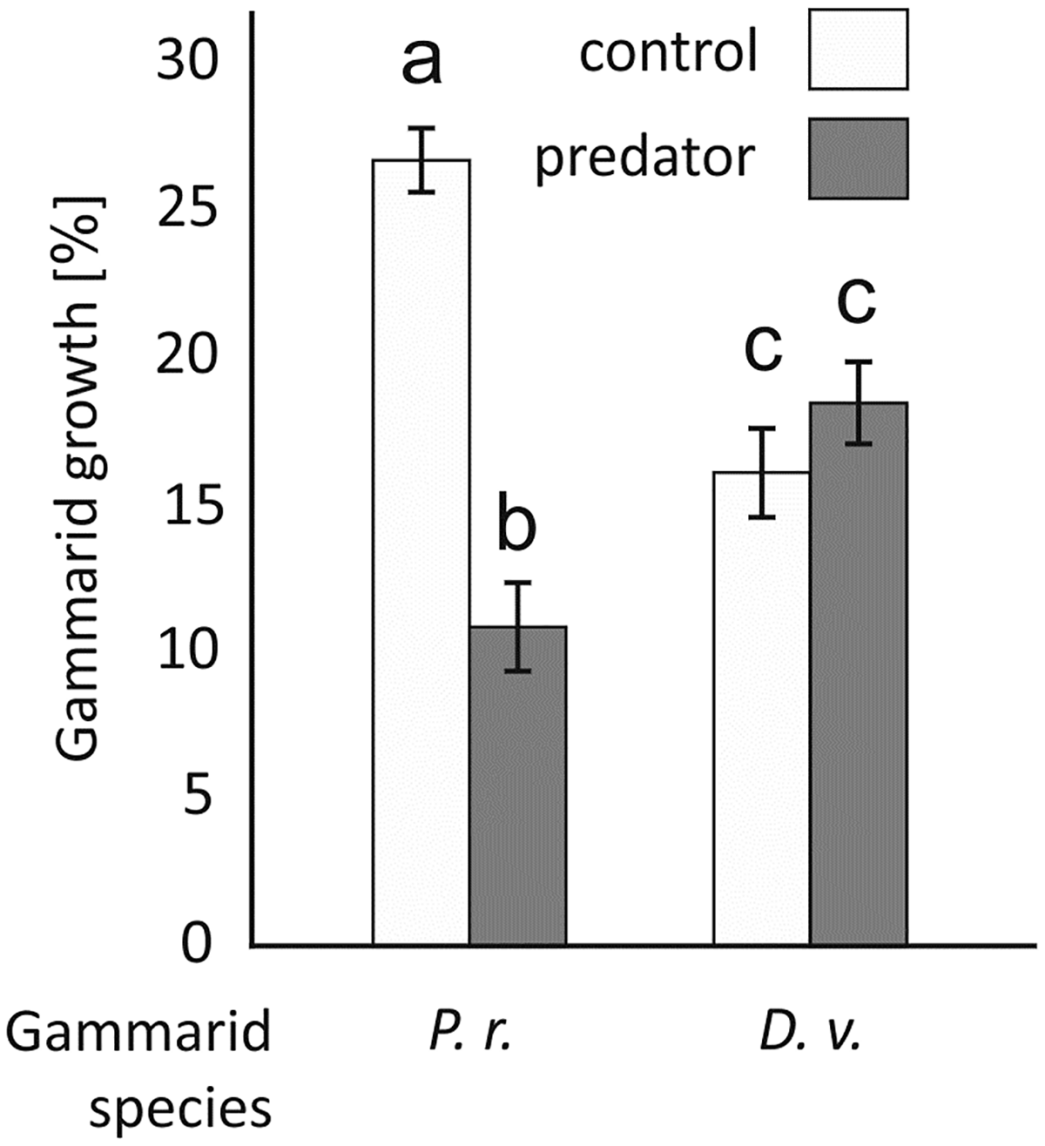
The effect of the predator presence on the mean 2-week growth of *Dikerogammarus villosus* (D.v.) and *Pontogammarus robustoides* (P.r.) expressed as a percentage of initial weight (Experiment III). Treatments labelled with the same letter do not differ significantly (P > 0.05) from one another. Error bars indicate standard errors of the mean.

Gammarid growth depended on gammarid species and predator presence, which resulted in a significant interaction in the ANOVA (Table 4). Under control conditions, *P*. *robustoides* exhibited faster growth than *D*. *villosus* but in the presence of fish the growth of *P*. *robustoides* was significantly reduced. The growth of *D*. *villosus* was not affected by the predators (Fig 5).

**Table 4:** Three-way nested ANOVA to test the effect of the tanks (random factor), gammarid species and presence of predators on the gammarid growth rate (Experiment III).

Effect	df	MS	F	P	η^2^
Predator (P)	1	0.265	4.853	0.035	0.127
Species (S)	1	0.033	0.609	0.441	0.018
Tank (nested in P x S)	28	0.057	1.554	0.047*	0.204
P x S	1	0.434	7.925	0.008*	0.192
Error	33	0.055			

(*) Asterisks indicate statistically significant effects.

η^2^ values indicate the effect sizes for particular terms.

## Discussion

The aim of experiment I was the determination of the inclination to take risk by gammarids during feeding, which can be an indirect indicator of their behavioural resistance to predator NCEs. We simulated a situation when gammarids were forced to search for food resources outside the shelter, thus increasing predation risk. Previous work indicated that the two, species differ from each other in their anti-predator responses [26] and activity patterns [44]. This difference could depend on their different morphological adaptations [37], as demonstrated in the case of armoured and not-armoured invertebrates [15]. Therefore, we predicted that the behavioural response exhibited by *D*. *villosus* would be weaker, since it has a harder exoskeleton [37] and a higher survival rate in the presence of predators [38]. However, contrary to our predictions, in the presence of predators both species significantly reduced their consumption, even when the distance to the feeding ground was short and capture risk low. Moreover, despite assumption of a more effective anti-predator mechanism, *D*. *villosus* bore a higher cost of the anti-predator response than the competing species. Generally, *P*. *robustoides* exhibited higher consumption than *D*. *villosus*, probably due to its higher activity [44] facilitating food localization, but also likely associated with higher energetic requirements. The availability of resources (in our study: the distance to the feeding ground) may alter the strength of NCE [45], because of the trade-off between the predation risk and foraging rewards [46]. However, in our study the response of gammarids was the opposite to that expected, as their reaction to predators was stronger under theoretically safer conditions, when food was located close to their shelters.

Reduced time spent in the open space in the predator presence suggests that the longer shelter utilization was the main factor responsible for the reduction of feeding. In accordance to the aforementioned consumption results, such a response induced by predation risk was observed only when food was in the direct proximity of the shelter. However, contrary to those results, the antipredator response of *P*. *robustoides* was stronger than that of *D*. *villosus*, which suggests that feeding efficiency is not always directly related to activity reduction. Gammarids exhibit strong association with shelters [36,42]. In the open area their defence abilities are limited and predation risk high [37,38]. Therefore, when forced to search for food located far from their shelters, gammarids could react as in a threat situation even without a direct predator signal. Nevertheless, they did spend more time in the open space when food was located further from the sheltered area, which shows that they compromised safety to obtain less available food. Activity reduction, owing to a switch to safer habitats is a common anti-predator response [47,48], which may result in the decrease in feeding, lower energy income and growth disturbance [11,49,50]. However, in the case of benthic invertebrates, the cost of such a response is also related to the flexibility of prey feeding and quality of the inhabited substratum. Both gammarid species are omnivores capable of feeding on both plant and animal matter [30,31,51]. *Dikerogammarus villosus* also commonly occurs in heterogeneous habitats, such as dreissenid colonies, which increase the availability of its food resources [52,53] and guarantee a higher safety level than other substrata [38]. In such a situation, animals do not need to leave their shelters and, despite movement reduction, have the opportunity to feed, limiting the potential cost of predator presence and simultaneously reducing predator efficiency.

To investigate theability of gammarids to feed in the predator presence, we conducted experiment II, testing their behaviour at a microscale and over a relatively short time period. In this experiment, gammarids had food in their direct vicinity and did not have to search for it, as in experiment I. In contrast to the results of experiment I, the predation risk did not limit the consumption of both gammarid species. Thus, the higher shelter utilisation and less efficient food searching was the main reason for the feeding reduction in experiment I. In contrast, when food was in the direct proximity of gammarids and consumption was not related to their activity, the amount of consumed food was not reduced. Theoretically, it was also possible that in experiment I the predation cue was stronger than in experiment II, as gammarids could also perceive visual and mechanical (mediated by water movements) signals from the fish present in the tank. However, chemical cues are claimed to be responsible for the predator detection by gammarids [26,41,43], given both the nature of aquatic environment and quality of their sensory organs. The dense and stiff mesh separating the fish from gammarids should have been sufficient to dampen water movements caused by relocating fish and their visual cues. Moreover, relatively simple gammarid eyes, consisting only of several dozen ommatidia, are unable to form sharp images and help in predator identification through the separating mesh [54].

Experiment I showed that activity reduction and higher shelter utilization were basic anti-predator responses of alien gammarids, which generated potential costs due to isolation from food resources. On the other hand, experiment II suggested that the observed NCE costs could be limited if prey chose an optimal habitat, guaranteeing the availability of food resources accessible without leaving the shelter. For benthic invertebrates, the substratum quality is a fundamental factor shaping habitat conditions, providing food [55], safe shelter [38] and protection from water movements [56]. Therefore, aquatic taxa are able to select optimal substrata [36,42] allowing for significant reduction of the NCE cost.

Experiments I-II showed short-time behavioural consequences of predator presence, which were quite similar for both gammarid species, despite differences between them in the effectiveness of their antipredator responses [26,28,35,37,38]. The opposite situation was observed for dreissenid mussels, where the stronger competitor, *Dreissena rostriformis bugensis* was found to exhibit weaker short- and long-time anti-predation responses than its congener *D*. *polymorpha* [57]. However, NCE costs are also associated with energy-costly long-term morphological and physiological responses, including disturbances of the immune defence system and/or increase in the activity of antioxidant defence system [58,59]. Such responses affect prey growth and reproduction due to the allocation of available resources to defence mechanisms [11,60]. To evaluate the long-term cost of the predator presence, we conducted experiment III, in which gammarids were exposed to predator cues for 2 weeks under unlimited food conditions. Our results demonstrated a significant reduction in the growth rate of *P*. *robustoides* in the presence of predators, whereas the growth of *D*. *villosus* remained unchanged. Neither of the gammarid species reduced their consumption when they did not have to leave their shelters to find food in experiment II. Thus, reduced consumption was not a likely reason for the observed growth disturbance of *P*. *robustoides* in experiment 3, contrary to the results of observations of damselfly larvae and bivalves exposed to predation risk [61,62]. Continuous predator presence induces chronic stress, which results in the disruption of cellular homeostasis and reduction of escaping abilities [63]. Under predation risk, prey species activate physiological compensatory mechanisms to limit the negative consequences of higher metabolic rate [61]. However, the efficiency of such mechanisms is limited and depends on many factors including time [61] food availability and prey sex [58].

Thus, *P*. *robustoides* seems to bear lower consequences of the predator presence when food is unavailable without leaving a safe shelter. However, when food is available in the direct proximity, *D*. *villosus* performs better in the presence of predators, exhibiting higher resistance to the consequences of chronic predation risk, probably due to more effective compensatory mechanisms or generally lower stress responses to the predator presence. It is also possible that *P*. *robustoides*, despite its higher (experiment I) or similar (experiment II) consumption compared to that of *D*. *villosus*, is not able to sustain its growth in the presence of predators in experiment III due to greater energetic losses induced by the higher stress generated by predation cues and/or generally higher energetic demands of this more active species. Probably, the anti-predatory resistance of *D*. *villosus* may be an effect of morphological and/or behavioural adaptations making it relatively less attractive prey item for benthivorous fish [37]. Further studies are needed to evaluate the effectiveness of chronic stress compensation in species characterized by different predator vulnerability.

Features making *D*. *villosus* such a successful invasive species have been discussed in previous literature [29,64]. However, research focused on anti-predator strategies exhibited by invasive species and linking them to invasion success is limited [28,37,38], and we are unware of previous research that assess NCE costs in the presence of predators. It seems likely that invasive potential can be correlated with NCE cost imposed by predators and invasive potential can be correlated. Although the short-term feeding responses of alien gammarids in our experiments I and II were rather similar irrespective of the effectiveness, of their anti-predator strategy, *Dikerogammarus villosus* was more resistant to long-term negative consequences of NCEs in experiment III. At the same time, this species is known for its relatively more efficient anti-predation strategy [37,38] and its invasion success, estimated by the larger invaded range and impact, is much greater [34,35]. However, to be effective, such a resistance requires appropriate habitat conditions. We have shown that optimal habitat choice, in particular the presence of food available without leaving sheltered locations, is crucial for reduction of the NCE costs in alien gammarids. Moreover, due to the observed interspecific differences of actual NCE costs, the predator presence may still increase the competitive advantage of *Dikerogammarus villosus* over other gammarids, even despite the reduction of interspecific competition [35].

## Supporting information

S1 File The list of datasets containing the data from Experiments I-III. (XLSX)
